# A Rare Phenomenon of Isaacs Syndrome: A Case Report

**DOI:** 10.7759/cureus.34150

**Published:** 2023-01-24

**Authors:** Arsh N Patel, Parth K Patel, Jaydip Desai, Srivikram Margam S, Katie Oakley, PJ Reddy

**Affiliations:** 1 Department of Research, Alabama College of Osteopathic Medicine, Dothan, USA; 2 Department of Research, George Washington University School of Medicine and Health Sciences, Washington, D.C., USA; 3 Department of Internal Medicine, Decatur Morgan Hospital, Decatur, USA

**Keywords:** anti-leucine-rich glioma-inactivated 1, caspr2, sudorrhea, myopathy, autoimmune myopathy, voltage gated potassium channel antibody, morvans syndrome, auto immune, isaacs syndrome, acquired neuromyotonia

## Abstract

We illustrate the case of a 71-year-old male who initially presented with sudden onset muscle weakness and ambulation difficulty. Following medication discontinuation and additional clinical studies, he failed to improve and was admitted to the hospital 11 weeks later. He had an associated 20-pound weight loss, sudorrhea, and muscle stiffness only when weight-bearing. A complete connective tissue cascade and a paraneoplastic panel were obtained. Clinical diagnosis of acquired neuromyotonia, or Isaacs syndrome (IS), was made, and he began experiencing significant improvement after intravenous steroid infusion. IS is a rare disease that has been poorly documented in the literature. There have only been a limited number of cases which are globally documented. One of the difficulties is a lack of definite autoantibody with which to correlate the disease; however, there has been some correlation linking the disease to voltage-gated potassium channels. Ultimately, the diagnosis should be driven by history and clinical presentation. The aim of this case report is to highlight a rare disease process and increase awareness among clinicians. We also describe the associated evaluation and recommended treatment for an optimal patient outcome.

## Introduction

Neuromyotonia, otherwise known as Isaacs syndrome (IS), is an autoimmune condition characterized by spontaneous and or continuous muscle fiber activity resulting from peripheral nerve hyperexcitability [1,2]. According to Orphanet, only 100-200 total cases of IS have been recorded across the 41 countries within their network [3]. Of the afflicted population, the majority are male at around 67% and the median age is 55 years; however, the incidence has been recorded in patients as young as 15 [2,4]. IS is classified under the umbrella of peripheral nerve hyperexcitability syndromes and is one of the most severe variants alongside Morvan syndrome; the milder presentations on this spectrum include benign fasciculation syndrome, which is known to have a bidirectional link with anxiety and cramp fasciculation syndrome [5,6]. IS and Morvan syndrome present with several muscle contraction abnormalities combined with dysautonomia, including hyperhidrosis, and pain [2]. A key aspect in differentiating the two disease entities is that IS does not have the characteristic neuropsychiatric involvement Morvan syndrome typically features [5].

Autoantibodies against voltage-gated potassium channels (VGKCs) have been seen in 45-50% of IS [7]. While there is still a debate on the specifics, the targets of these autoantibodies most commonly associated are contactin-associated protein 2 (CASPR2), leucine-rich glioma-inactivated protein (LGI1), and contactin-2 [2,8]. The overlap of antibody targets with several other conditions like Morvan syndrome indicates other targets may be involved in IS etiology. The VGKC transmembrane channel, which is central to the pathology of a large number of cases of Morvan syndrome and IS, complexes with CASPR2, LGI1, and contactin-2 proteins [2,4,9], with CASPR2 being the most commonly targeted protein [9]. The widespread localization of LGI1 and CASPR2 in the central nervous system has implications on the pathophysiology of Morvan syndrome as well as the CNS effects seen in some cases of IS; the proteins are very commonly found in the neurons of the thalamus, hypothalamic, locus coeruleus, and the raphe nuclei [9]. The hypothalamic orexin neurons and the locus coeruleus are both involved in the control of arousal, wakefulness, and the sleep cycle; dysregulation of these pathways could explain the insomnia commonly observed in patients [9]. As the locus coeruleus is the synthetic site of norepinephrine, dysregulation in this area could account for the dysautonomia (i.e. hyperhidrosis, tachycardia, hemodynamic instability) often seen in both IS and Morvan syndrome. Both LGI1 and CASPR2 are also found in the hippocampus, which could imply potential deleterious effects on memory formation. 

The immunological aspect of IS has been associated with paraneoplastic mechanisms given that malignancies are reported in 21-25% of IS patients [7]. This may indicate that antibodies formed in response to tumor-associated antigens are initiating an autoimmune reaction via cross-reactivity with VGKC epitopes [10]. Our paper demonstrates a rare case of acquired neuromyotonia in an elderly male with limited risk factors for development. We aim to contribute to the limited documented literature as well as to increase awareness of the associated autoimmune phenomena and the established management and treatment guidelines of IS.

## Case presentation

A 71-year-old male presented with the primary complaint of a decrease in his routine activities and ambulation difficulties associated with weakness and cramping. His medical history includes coronary artery disease, gastroesophageal reflux with Barrett’s esophagus, and prior lacunar stroke. He denied any current or past tobacco use but had used alcohol in the past. Extensive work-up showed an elevation of creatinine kinase (CK) at 748 Units per Liter (U/L) and aldolase at 8.2 U/L, but negative Lyme disease and Rickettsia antibodies. It was believed to be caused by atorvastatin-induced myopathy, so his medication was discontinued with a scheduled follow-up for re-evaluation of symptoms. Despite medication discontinuation, his muscle weakness and gait troubles persisted. Neurologic consultation and electromyography (EMG) studies suggested findings consistent with myopathy attributed to polymyositis. A left thigh biopsy completed by his neurologist showed denervation atrophy.

**Figure 1:**
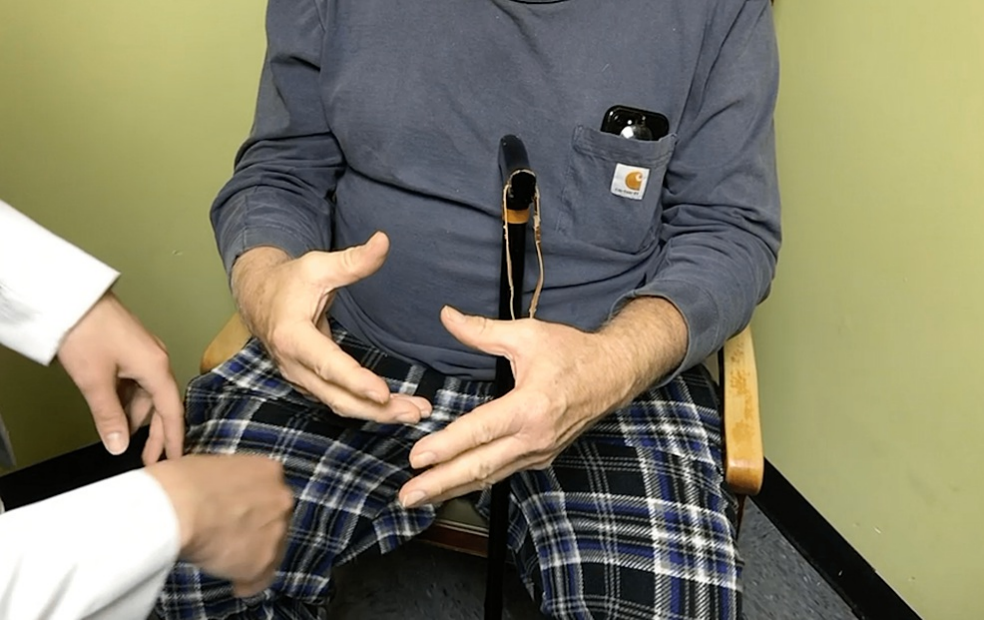
Physical Examination Finding Involuntary contractions causing flexion at the metacarpal phalangeal joint and extension at both interphalangeal joints.

Eleven weeks later, the patient was admitted directly from the emergency department after waking up in the morning unable to stand and walk due to lower extremity stiffness. The stiffness present in the legs was present only during weight-bearing and resolved when non-weight bearing. When attempting to relax his hands he had involuntary contractions causing flexion of the fingers at the metacarpal phalangeal joint and extension at both interphalangeal joints (Figure 1). Additionally, he reported hyperhidrosis, most prominently during sleep which required changing his clothes and bedding every one to two hours. He associated an unintentional 20-pound weight loss during this period. He denied any trouble chewing, swallowing, slurred speech, loss of appetite, fever, chills, visual disturbances, facial asymmetry, abnormal facial contractions, or ptosis. Computed tomography (CT) scans of the chest, abdomen and pelvis were performed to rule out any paraneoplastic disease, all of which were negative. Extensive and serial laboratory studies included a paraneoplastic antibody panel (Table 1), connective tissue cascade (Table 2), and thrombophilia workup (Table 3). The thorough workup showed elevated muscle acetylcholine receptor antibodies, anti-double-stranded DNA antibodies, and positive antinuclear antibodies. The working diagnosis consisted of myopathy, polymyositis, stiff person syndrome, myasthenia gravis (MG), and IS.

**Table 1:** Paraneoplastic Panel nmol/L = nanomoles per liter

Laboratory Study	Value	Reference
Hu (ANNA-1) Antibody	Negative	Negative, titer <1:240
Ri (ANNA-2) Antibody	Negative	Negative, titer <1:240
Anti-Neuronal Nuclear Antibody 3 (ANNA-3)	Negative	Negative, titer <1:240
Anti-Glial Nuclear Antibody 1 (AGNA-1)	Negative	Negative, titer <1:240
Purkinje Cell Cytoplasmic Antibody 1 (PCA-1)	Negative	Negative, titer <1:240
Purkinje Cell Cytoplasmic Antibody 2 (PCA-2)	Negative	Negative, titer <1:240
Purkinje Cell Cytoplasmic Antibody, type Tr (PCA-Tr)	Negative	Negative, titer <1:240
Amphiphysin Antibody	Negative	Negative, titer <1:240
Collapsin Response-Mediator Protein-5 IgG (CRMP-5-IgG)	Negative	Negative, titer <1:240
Striational (Striated Muscle) Antibody	Negative	Negative, titer <1:240
P/Q-type Voltage Gated Calcium Channel Antibody	0.00 nmol/L	≤0.02 nmol/L
N-Type Voltage Gated Calcium Channel Antibody	0.00 nmol/L	≤0.03 nmol/L
Acetylcholine Receptor (muscle) Binding Antibody	0.30 nmol/L	≤0.02 nmol/L
Acetylcholine Receptor Ganglionic Neuronal Antibody	0.01 nmol/L	≤0.02 nmol/L
Neuronal (V-G) Potassium Channel Antibody	0.00 nmol/L	≤0.02 nmol/L
GAD 65 Antibody	0.00 nmol/L	≤0.02 nmol/L
Anti-Mitochondrial Antibody	<0.1 Units	<0.1 Units

**Table 2:** Connective Tissue Cascade [IU]/mL = International Units per milliliter

Laboratory Study	Value	Reference
Antinuclear Antibody (ANA)	Positive, 1:160 Speckled	Negative
Double-Stranded DNA Antibody	197 [IU]/mL	<200 [IU]/mL
Chromatin Antibody	<0.2 [IU]/mL	<1.0 [IU]/mL
Smith (SM) Antibody	<0.2 [IU]/mL	<1.0 [IU]/mL
Ribonucleoprotein (RNP) Antibody	0.2 [IU]/mL	<1.0 [IU]/mL
SM-RNP Antibody	<0.2 [IU]/mL	<1.0 [IU]/mL
Ribosomal Protein Antibody	<0.2 [IU]/mL	<1.0 [IU]/mL
Anti-Ro/SS-A Antibody	<0.2 [IU]/mL	<1.0 [IU]/mL
Anti-La/SS-B Antibody	<0.2 [IU]/mL	<1.0 [IU]/mL
Anti-anti-histidyl transfer RNA (tRNA) synthetase (anti-Jo-1) Antibody	<0.2 [IU]/mL	<1.0 [IU]/mL
Anti-Topoisomerase I (SCL-70) antibody	<0.2 [IU]/mL	<1.0 [IU]/mL
Anti-Centromere B Antibody	<0.2 [IU]/mL	<1.0 [IU]/mL
Anti-Proteinase 3 Antibody (C-ANCA)	Negative	Negative
Anti-Myeloperoxidase Antibody (P-ANCA)	Negative	Negative
Anti-Cyclic Citrullinated Peptide	<15.6 Units	<20.0 Units

**Table 3:** Thrombophilia Workup Results ug/mLFEU = micrograms per milliliter of fibrinogen equivalent units; s = seconds; % = percent activity of enzyme in serum

Laboratory Study	Value	Reference
D-dimer	3.30 ug/mLFEU	0.0-0.52 ug/mLFEU
Lupus Anticoagulant Prothrombin Time	48.0 s	31.5-48.6 s
Protein C functional Activity	148%	64-154%
Protein S Activity	96%	77-143%
Antithrombin III activity	87%	88-140%
Factor V Leiden mutation	Negative	Negative
Prothrombin G20210A mutation	Negative	Negative

Despite a negative VGKC antibody test, clinical presentation suggested that the most probable diagnosis was sudorrhea with neuromyotonia (IS). During his hospitalization, our patient was started on high-dose one-gram intravenous methylprednisolone in normal saline at 200 milliliters per hour and reported significant improvement in his symptoms. His repeat CK and aldolase levels were normalized to 43 U/L and 4.2 U/L, respectively, prior to discharge. Upon discharge, he was tapered to 60 milligrams (mg) of oral prednisone daily, life-long rivaroxaban, a maintenance dose of 200 mg carbamazepine three times daily, and therapeutic plasma exchange therapy and was given a referral for a neuromuscular junction clinic. The treatment protocol also included the discontinuation of the patient’s statin and pyridostigmine. 

## Discussion

We present an elderly male with several comorbid conditions who was eventually diagnosed with acquired neuromyotonia, also known as IS. Similar to several other neuromuscular diseases, the phenomenon of neuromyotonia begins with progressive muscle stiffness leading to widespread fasciculations. A key component involved in the workup for diagnosis is continuous muscle involvement at rest on EMG [7]. In the setting of our case, the EMG performed did not demonstrate any similar outcomes. This may be perhaps due to the certain medications involved in his plan of care that led to myopathy. Previous literature has also demonstrated that a single EMG study can be susceptible to temporal sampling error, as patients who did not have the characteristic doublet, triplet, or multiplet discharges on their first EMG developed these findings on their second or third EMG study [5]. Another finding that may be seen is myokymia, with which the patient in our case did not complain of or present. 

IS has autoimmune components that are essential for diagnosis; autoantibodies are identified in about 45-50% of the affected population [7]. These autoantibodies include VKGCs, CASPR2, and LGI1. While these autoantibodies may be seen in multiple overlapping disorders, it is important to test for them in the clinical setting if suspicion is high. However, there is no specific correlation between autoantibody phenotype and symptom involvement. Furthermore, IS may have associated paraneoplastic disease involvement in approximately 21-25% of total cases [7]. CASPR2 antibodies have been documented with thymoma in about 20% of total cases. A case published in 2020 by Kim et al. highlighted the diagnosis of IS in a patient with MG who was undergoing chemotherapy and radiation treatment for thymic carcinoma [11].

Currently, there are no Food and Drug Administration-labeled medications specifically for use in the treatment of IS. Phenytoin, carbamazepine, and gabapentin have all shown efficacy with initial symptom relief in acquired neuromyotonia [12]. Because acquired neuromyotonia has an autoimmune association, treatment may also include plasmapheresis. If unsuccessful, additional therapy with intravenous immunoglobulin may provide some benefit but has not been well documented in the literature. It is also rational to begin oral steroid therapy with prednisolone, with eventual taper, and azathioprine or methotrexate to further boost immunosuppression. One case of IS reported in October 2022 demonstrated response to rituximab after initial treatment with many of the agents listed above was refractory [13]. 

Morvan syndrome is inclusive of IS; it is characterized by the classic triad of neuromyotonia, hyperhidrosis, and encephalopathy; encephalopathy is the factor which distinguishes Morvan syndrome from IS [14]. In Morvan syndrome, patients may present with hallucinations, confusion, agitation, and insomnia. As with IS, Morvan syndrome is often an autoimmune process associated with VGKC antibodies. Studies looking at patients with neuromyotonic symptoms have found that a significant minority of IS patients had some degree of central nervous system involvement, with the most severe cases classified as Morvan syndrome requiring antipsychotic treatment; the patients with Morvan syndrome also happened to have the highest VGKC antibody titers, suggesting a potential quantitative relationship between antibody titer and the manifestation of Morvan syndrome as an exaggerated version of IS [5]. While it is unclear whether the mild anxiety and insomnia seen in the IS patients are secondary to the psychological effect of suffering from the disease itself versus being a result of the same pathophysiological process underlying the neuromuscular symptoms, the formerly stated findings do establish a potential link [5]. This is supported by a lack of cerebrospinal fluid antibodies found in Morvan syndrome patients, suggesting that circulating serum antibodies are primarily responsible for the symptoms, akin to IS, further lending credence to the idea of Morvan syndrome existing on the same pathophysiological spectrum as a severe extension of IS [5]. 

IS can be paraneoplastic; it is most commonly associated with thymomas and small-cell lung cancers [9,15]. In one study, 20% of patients classified with neuromyotonia according to doublet, triplet, or multiplet EMG discharges had thymoma, while 10% had lung cancer. Even in the group without the characteristic EMG findings, which would have been classed as having cramp fasciculation syndrome, less severe than neuromyotonia on the axis of peripheral nerve hyperexcitability syndromes, 11% had thymoma and 6% had some form of lung cancer [5]. It is also of note that autoimmune diseases occurred more frequently than would be expected by pure chance alone in both of the groups analyzed; the most common association was with MG and antibodies against the acetylcholine receptor (AchR) [5]. It is presently unclear how much of this is linked to the coexistence of thymoma in these patients, as thymoma is often associated with MG as well, versus the pathogenesis of IS itself [16]. These findings provide further implicit evidence for the shared mechanisms between different manifestations of peripheral nerve hyperexcitability and underscore the need to screen patients with this constellation of symptoms thoroughly to rule out underlying neoplasia. While there is no fixed guideline in this regard, whole-body magnetic resonance imaging (MRI) may be considered an aggressive screening measure [9]. Chest CT and chest MRI should also be strongly considered in high-risk patients such as elderly smokers with late-onset disease [5].

## Conclusions

This case reported an elderly man with no known history of neoplastic or other autoimmune disease who was given a clinical diagnosis of IS and successfully treated with high-dose steroids, rivaroxaban, carbamazepine, and plasma exchange therapy. Neuromyotonia, or IS, is a rare autoimmune condition that is characterized by peripheral nerve hyperexcitability, dysautonomia, anxiety, and pain. While it is unlikely that all the autoantibody targets of this disorder have been characterized, some of its recognized targets include CASPR2, LGI1, and contactin-2. Neuromyotonia is also often associated with neoplasms such as thymoma as well as autoimmune conditions including MG. This case serves to highlight a rarely reported disease process and to underscore effective treatment options for IS.

## References

[1] Maddison P (2006). Neuromyotonia. Clin Neurophysiol.

[2] Bashford J, Chan WK, Coutinho E, Norwood F, Mills K, Shaw CE (2021). Demystifying the spontaneous phenomena of motor hyperexcitability. Clin Neurophysiol.

[3] (2022). Issacs Syndrome. https://www.orpha.net/consor/cgi-bin/OC_Exp.php?lng=EN&Expert=84142.

[4] (2022). Acquired Neuromyotonia. https://rarediseases.org/rare-diseases/acquired-neuromyotonia/.

[5] Hart IK, Maddison P, Newsom-Davis J, Vincent A, Mills KR (2002). Phenotypic variants of autoimmune peripheral nerve hyperexcitability. Brain.

[6] Taylor P (2005). Issacs’ syndrome (autoimmune neuromyotonia) in a patient with systemic lupus erythematosus. J Rheumatol.

[7] Park SB, Thurbon R, Kiernan MC (2020). Isaacs syndrome: the frontier of neurology, psychiatry, immunology and cancer. J Neurol Neurosurg Psychiatry.

[8] Nakazato T, Tsuji Y, Kanai K (2018). Isaacs syndrome: a slow potassium channelopathy caused by autoantibodies?. Clin Neurophysiol.

[9] Epure DA, Ioghen MR, Roza E, Teleanu RI (2020). A case report of Morvan syndrome, the unique clinical pattern of a rare disease. Ro J Neurol.

[10] Binks SN, Klein CJ, Waters P, Pittock SJ, Irani SR (2018). LGI1, CASPR2 and related antibodies: a molecular evolution of the phenotypes. J Neurol Neurosurg Psychiatry.

[11] Kim H, Lim YM, Lee EJ, Kim HW, Ahn HS, Kim KK (2020). Anti-CASPR2-antibody-positive Isaacs' syndrome presenting with myokymia, neuropathic pain, and hyperhidrosis. J Clin Neurol.

[12] Ahmed A, Simmons Z (2015). Isaacs syndrome: a review. Muscle Nerve.

[13] Horiuchi K, Kudo A, Inoue T, Fujii S, Oshima Y (2022). Rituximab was effective in relieving symptoms of Isaacs syndrome: a case report. Cureus.

[14] Barber PA, Anderson NE, Vincent A (2000). Morvan's syndrome associated with voltage-gated K+ channel antibodies. Neurology.

[15] Li KC, Liao MF, Wu YR, Lyu RK (2022). Isaacs' syndrome as the initial presentation of malignant thymoma and associated with double-positive voltage-gated potassium channel complex antibodies, a case report. BMC Neurol.

[16] Romi F (2011). Thymoma in myasthenia gravis: from diagnosis to treatment. Autoimmune Dis.

